# 

**Published:** 1890-11

**Authors:** 


### Communications.

### THE CALIFORNIA STATE BOARD OF DENTAL EXAMINERS, AND ITS INFLUENCE ON DENAL EDUCATION.

*Editor of the American Journal of Dental Science:*

An essay was read before the Southern California Odontological Society at the fifth annual meeting, October 14th, 1890, by Jno. C. McCoy, D. D. S., of Santa Ana, Cal.

The motto of American dentists has ever been "Excelsior," never satisfied with past wonderful improvements, ever pressing onward and upward to a proficiency not even dreamed of by the most advanced of the past generation.

Dental Colleges have aided largely in raising the standard of the profession. In order to compel all to attain sufficient knowledge before practicing, but most of all to protect an innocent and over-confiding public, our Legislatures have enacted laws to regulate the practice of dentistry.

The title of our California law as approved March, 1885, is as follows: "An act to insure the better education of the practitioners of dental surgery, and to regulate the practice of dentistry in the State of California."

The Governor appointed seven practicing dentists of our State to carry out the provisions of our dental law.

Now be it understood that the object for which this "State Board of Dental Examiners" was created, was "to insure the better education of the practitioners of Dental Surgery."

In other words, they were intrusted by our State with the important mission of protecting her citizens from the malpractice of unqualified practitioners of dentistry, that is, so far as the qualifications of those who applied to them for certificates to prac̄tice dentistry in the State of California were concerned.

This Board of Dental Examiners has been meeting for examination of candidates for five years. Their work is open to inspection of the public, and now we want to inquire what has been the influence of this Board on dental education.

1. The examinations have been theoretical and not at all practical. The first course student of a dental college with scarcely any practical knowledge is placed side by side with the experienced practitioner, and both subjected to a written examination without an opportunity to show by clinical demonstration their practical knowledge.

No member of this State Board of Dental Examiners could state on oath whether any applicant could properly perform even the simplest operation; one young man who successfully passed the examination boasted that up to that time he had never made a set of artificial teeth.

The position of our State Board of Dental Examiners is perhaps unique, it probably being the only one in the United States opposed to the better education of the dental practitioners.

Knowing that the above is true, we would like to ask, how much this act insures the better education of the practitioners of dental surgery ?

2. We will examine the attitude of the State Board of Dental Examiners towards the Dental Department of our State University. Our State Dental College is worthy the encouragement and patronage of every dentist of this State; and is really doing all that is being done educationally on this Coast. This College has adopted a high standard for its graduates. To graduate from our State Dental College means a thorough training in all branches of dentistry.

Now this same State Board of Dental Examiners is antagonistic to the Dental College. A State institution and working under the same authority. In short they grant certificates to practice to students who have failed to pass required examinations in the college; thus they send forth unqualified practitioners, deceiving the people, being clothed with all the legal rights of a competent and educated dentist.

Please remember the purpose for which this Board was created: To insure the *better education* of the practitioners of dental surgery.

They have not only failed to fulfill their mission, but have been and are obstructionists, and have prevented the very thing they were appointed to promote.

3. Has this Board co-operated with the profession of the State to secure the end for which they were appointed, viz: To insure the *better education* of the practitioners of dental surgery?

This Society believing that the law regulating the practice of dentistry could be improved, and that the State Board of Dental Examiners was guilty as charged in a former part of this paper, appointed a committee to confer with a like committee of the "State Dental Society" with reference to amending the dental law so as to limit the power of the Board to the examination of diplomas.

The State Dental Society, (the leading members of which are on the Board of Dental Examiners,) referred our petition to the said Board, a distinction without a difference, who, presumably fearing their power and authority would be limited by such a change in the law, paid no attention to our requests, and by so doing refused to co-operate with those interested in insuring the better education of the practitioners of dental surgery.

Our evidence is all given, our argument is made. The case is before you and the profession of our great State shall be the jury. We will leave them to decide the influence our State Board of Dental Examiners is having on dental education.

GEO. E. PURNELL, *Cor. Sec'y.*

### THE KANSAS CITY ACADEMY OF MEDICINE.

KANSAS CITY, MO., NOV., 14th, 1890.

*Editor of the American Journal of Dental Science :*

The "Kansas City Academy of Medicine," incorporated last July, now has all the active young medical men of Kansas enrolled as "Fellows." In order to carry out the object of the Academy, a good Medical Library was indispensable and the majority of the "Fellows" being young men of limited means we reached out and asked our teachers and authors of medical books, as children ask their parents, for help.

Almost every author sent copies of their books and as a result, the Academy now has about 150 volumes of standard medical works.

The publishers of the leading medical journals, (about 100,) have each promised to send copies of their journals to to the library rooms gratuitously. The liberality of our teachers and authors has placed us on a good financial basis and we have secured permanent quarters.

If you will contribute to the Library we will be very grateful. Yours truly,

JAMES P. PARKER, M. D.,
*Chairman of Library Committee.*

CHAS. B. HARDIN, M. D., *Sec'y.*

### Editorial, Etc.

### To Readers and Correspondents.

Communications intended for publication, and exchange journals and papers, should be addressed to the Editor of The American Journal of Dental Science, No. 845 N. Eutaw St. Baltimore, Md. All letters relating to the business of the Journal, or containing cash remittances, to be directed to Messrs. Snowden & Cowman, No. 9 W. Fayette Street, Baltimore, Md. Articles for publication should be received by the Editor at least two weeks previous to the first of the month on which the number in which it is designed for them to appear, is issued.

☞ The American Journal of Dental Science will contain fifty pages of reading matter in each number, making a volume of about Six Hundred pages,

EXCLUSIVE OF ADVERTISEMENTS.

